# Sensor and Methodology for Dielectric Analysis of Vegetal Oils Submitted to Thermal Stress

**DOI:** 10.3390/s151026457

**Published:** 2015-10-16

**Authors:** Sergio Luiz Stevan, Leandro Paiter, José Ricardo Galvão, Daniely Vieira Roque, Eduardo Sidinei Chaves

**Affiliations:** 1 Federal University of Technology - Paraná (UTFPR) - Electrical Engineering Graduate Program - PPGEE-UTFPR-PG; Av. Monteiro Lobato, km 04 - s/n°, Ponta Grossa 84016-210, Brazil; E-Mails: leandro.paiter@gmail.com (L.P.); jrgalvao@utfpr.edu.br (J.R.G.); 2 Federal University of Technology- Paraná (UTFPR) - Chemical Engineering Academic Department - DAENQ-UTFPR-PG; Av. Monteiro Lobato, km 04 - s/n°, Ponta Grossa 84016-210, Brazil; E-Mails: danielyroque@gmail.com (D.V.R.); eschaves@utfpr.edu.br (E.S.C.)

**Keywords:** dielectric constant, capacitive sensor, oil vegetal, recycle

## Abstract

Vegetable oils used in frying food represent a social problem as its destination. The residual oil can be recycled and returned to the production line, as biodiesel, as soap, or as putty. The state of the residual oil is determined according to their physicochemical characteristics whose values define its economically viable destination. However, the physicochemical analysis requires high costs, time and general cost of transporting. This study presents the use of a capacitive sensor and a quick and inexpensive method to correlate the physicochemical variables to the dielectric constant of the material undergoing oil samples to thermal cycling. The proposed method allows reducing costs in the characterization of residual oil and the reduction in analysis time. In addition, the method allows an assessment of the quality of the vegetable oil during use. The experimental results show the increasing of the dielectric constant with the temperature, which facilitates measurement and classification of the dielectric constant at considerably higher temperatures. The results also confirm a definitive degradation in used oil and a correlation between the dielectric constant of the sample with the results of the physicochemical analysis (iodine value, acid value, viscosity and refractive index).

## 1. Introduction

Oils and fats are water-insoluble substances of animal or vegetable origin more commonly formed by triglyceride esters, which result from the esterification between the glycerol and the fatty acids. At room temperature, the triacylglycerol have a consistency between liquid and solid. When solid, the triacylglycerol is called fat; and when liquid, it is known as oil. Besides the triacylglycerol, oils contain several components in lower proportion, such as mono and diglycerides, free fatty acids, tocopherol, proteins, sterols and vitamins [1,2].

Heating can modify the properties of oils and fats. These alterations can be classified as (i) self-oxidation: oxidation that occurs under 100 °C [3,4,5]; (ii) thermal polymerization: oxidation that occurs between 200 °C and 300 °C in the absence of [3,4,5]; (iii) thermal oxidation: oxidation that occurs in the presence of oxygen at high temperature (oxy-polymerization) [3,4,5]; (iv) physical: alterations that occur in the physical properties [3,4,5]; (v) nutritional: changes in the physiological and nutritional aspects of oils [3,4,5]; (vi) chemical: hydrolysis of the triglycerides, resulting in the release of fatty acids, glycerin, and mono and diglycerides; oxidation, which occurs in the fatty acids with double bonds; and polymerization with extensive condensation of fatty acid polyunsaturated at high temperatures for extended periods [3,4,5].

During the prolonged heating of the vegetable oil, the following occurs: polymerization process of the triacylglycerol molecules, resulting in more viscosity and a higher acidity of the oil; oxidation and hydrolysis resulting in sensorial and physical-chemical changes; and deterioration of the oil leading to an increase in conjugated dienes and trienes from unsaturated bonds, which increases the levels of thiobarbituric acid, peroxides, iodine as well as the refraction and viscosity indexes of the triacylglycerol molecule [6].

The quality of the vegetable oil exposed to a heating process may be determined through the analysis of the acidity, refraction and iodine indexes.

The acidity index is defined as the quantity (mg) of potassium hydroxide needed to neutralize the free acids that exist in one gram of oil or fat. The oil’s conservation state is deeply related to the nature and quality of the raw material, its processing and, particularly, with the storage conditions, as the decomposition of glycerides is accelerated when exposed to heating and lighting, while the rancidity is almost always followed by the formation of free fatty acid [7,8].

The iodine index is the quantity, in grams, of the iodine absorbed by 100 g of fat or oil. It provides the measurement of the degree of unsaturation of the fats extracted with ether or, yet, the measurement of the degree of unsaturation of the fatty acids found in the fat; the iodine can be quantitatively introduced in the double bonds of unsaturated fatty acids and triglycerides. This is the reason why the greater the unsaturation of a fatty acid, the greater its capacity to absorb iodine and, consequently, higher the index [8,9].

The refraction index of a medium is defined as the ratio between the speed of light in the vacuum and the speed of light in the medium. This quantity is directly related with the density of the medium; the higher the density, the lower the speed of light due to the photons interaction with the medium’s particles. Alterations in the refraction index of samples of vegetable oil before and after heating may reveal changes in the density of these samples due to the polymerization process [8].

As it results from the enzymatic hydrolysis, the acidity reveals the conservation state of the vegetable oil. The rancidity that results from this process has been followed by the formation of free fatty acids. This is why the acidity depends on the nature, quality, purity, processing, and, most importantly, the conservation state of the vegetable oil [10,11,12].

The quality of the vegetable oil that is consumed in a diet can increase the amount of fat in the adipose tissue, influence the body weight and, consequently, interfere in the development of non-transmissible chronic disease. In general, the saturated fatty acids tend to increase the cholesterol levels in the blood in every lipoprotein fraction. On the other hand, the consumption of foods that are sources of Polyunsaturated fatty acids, especially omega-3 and omega-6, is associated with a lower risk to develop several diseases, such as atherosclerosis and cardiovascular disease [12,13]. High consumption of this food in the fried form results in possible health damages, such as: pre-disposition to arteriosclerosis and mutagenic or carcinogenic action, due to the high toxicity of the products formed during the frying process, which are ingested and absorbed by the human body [14,15,16].

The analysis of the quality of the vegetable oil that has been exposed to a heating process involves the determination of a series of chemical indexes [7,8,9]. In this study, we propose an analysis of the vegetable oil’s quality, evaluating the acidity, refraction, iodine and viscosity indexes. Based on this approach, we propose an investigation to correlate the physical-chemical characteristics of the soybean oil used to fry in order to define a single unit of measurement that can qualify the condition this oil. To accomplish this goal, we used a capacitive sensor with the purpose of quantifying the level of deterioration of the edible vegetable oil as a result of repeated heating. The capacitive sensor was designed to evaluate this oil’s dielectric constant in relation to the use of heat. The sensor has helped us to observe the possible variations of the dielectric constant, the vegetable oil in this case, increasing its temperature and, therefore, stimulating a process of frying and re-using the oil (heating and cooling of the vegetable oil).

Some recent studies have reported the use of different techniques to assess quality of oils, such as, for example: colorimetric reactions [17], measurement of the total polar compound (TPC) [18,19,20,21] and near-infrared spectroscopy [22].

In this work, a capacitive sensor is used to correlate the dielectric constant of the oil with physico-chemical parameters, in particular, to evaluate it according to the temperature variation. This study shows that application of cycles of heating or continuous temperature changes the characteristics of the oil. Additionally, we can see that the application of temperature can work as an amplification of the dielectric constant, facilitating the evaluation of the state of a sample of oil.

In [23], a preliminary study was carried out showing that the behavior of dielectric constant of a soybean oil is amplified as a function of temperature.

Once qualified an oil used in relation to their physico-chemical changes due to temperature applied, this can be classified in terms of the possible destinations of this oil, such as soap making or obtaining biodiesel. In the last one, the transesterification method to be used is determined by the oil quality, and an acidity index smaller than 0.5 is desired in the function of the less expensive method [24].

## 2. Materials and Methods

### 2.1. Capacitive Sensor

**Figure 1:**
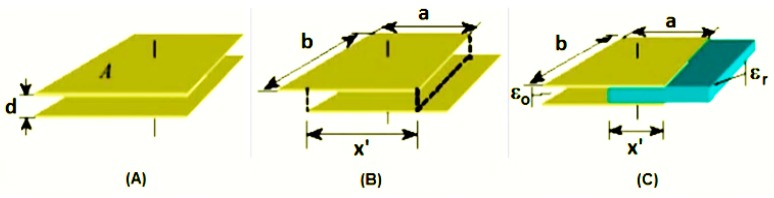
Illustration of a Capacitive Sensor of Parallel Plates, where (**a**) indicates a possible variation in the distance between the plates; (**b**) indicates a possible variation of the common surface; and (**c**) the alteration of the dielectric element.

A capacitive sensor is a device whose physical characteristics determine the value of its capacitance, according to the design at Figure 1. The capacitance value is altered when: there is an alteration of the separation distance between the plates “*d*”; or when there is a sliding of one of the plates, altering the common area “*a.b → x*ꞌ*.b*” between its surfaces, or still, when there are alterations of the dielectric element “ε_o_ *→* ε_r_” (partial or total exchange of the alterations element’s properties) between the plates. Figure 1 illustrates these possibilities.

The capacitance relates to the electrical energy storage capacity between the plates of a capacitor when they are submitted to a difference in potential. When we keep constant the overlapping area between the plates and the separation distance between them, the evaluation of capacitance allows us to characterize the dielectric element kept between its plates. It is also possible to determine the behavior of a dielectric element when this is subject to alterations in temperature, pressure, humidity, *etc.*

Equation (1) can be used to determine the capacitance (C) between the two parallel plates (electrodes) of a capacitor, where ε_r_ is a relative dielectric constant that depends on the dielectric material, ε_0_ is the constant of dielectric permissiveness in the vacuum (8.85 × 10^−12^∙C^2^∙N^−1^∙m^−2^), “A” is the plate’s common area, and “d” is the separation distance between the plates [25].

(1) $$C=\varepsilon_{r}\cdot \varepsilon_{0}\cdot \frac{A}{d}+{\text{C}}_{\text{a}}$$

where C_a_ is the Capacitance from effect of fringing of the field lines. Many studies present that C_a_ is dependent of the geometric relation of the width and a nominal gap and its influence is smaller than 5% if the geometric relation is bigger than 50 [26,27].

There are different methods to convert the capacitance variation of a sensor to another electric entity, which makes it possible to record and facilitate its monitoring. The most frequent methods are:

(a) Measurement of capacitive impedance (given by 1/[2πfC]), monitoring the voltage over a resistor connected in series to the capacitor and an AC power source. When the capacitance increases, there is a reduction in the impedance of the capacitor and an increase in the voltage of the resistor. The increase is not linear with the capacitance, but it can be approximately linear for low values of ohmic resistance [28].
(b) Measurement through a capacitive bridge fed by an alternate AC source (also non-linear). The advantage is that there is no alteration of the exit signal resulting from frequency changes. When adding an operational amplifier, it is possible to increase the exit signal proportionally to the capacitance [29].
(c) Conversion of the sensor’s capacitance into a square wave signal with proportional frequency. Normally, an electronic oscillator circuit (as CI CA555, for industrial applications) can be used in a monostable configuration. The exit’s frequency depends on the sensor’s capacitance [30].

### 2.2. Materials and Methods Used to Build the Sensor

The purpose of this study is to analyze the dielectric characteristics of vegetable oil samples placed as the dielectric medium between the plates of a capacitive sensor. The alterations in the dielectric properties of the sample related to the temperature change will be monitored and correlated with the most common physical-chemistry analyses applied to vegetable oil quality evaluation. The sensor designed consists of eight parallel plates, screwed side by side and insulated. In the experimental arrangement, the sensor was submerged in a recipient containing vegetable oil. In first moment, we use a glass recipient, and compare the results with rehearsals using an aluminum recipient and obtained very similar results. Therefore, we opted for the recipient aluminum to allow chemical bonds during heating, imitating real conditions of use and conditions of heated oils (for frying). The lower base of the sensor was maintained at a distance of 3.0 cm from the bottom of the base of the recipient while the upper base was maintained at 1.0 cm below the oil surface.

During measurements, it was possible to investigate the dielectric constant variation in relationship to the alteration in the capacitance value recorded by the sensor. For each heating cycle, we collected a sample of the material for further chemical analysis and, consequently, a possible correlation with the dielectric values that were found.

**Figure 2:**
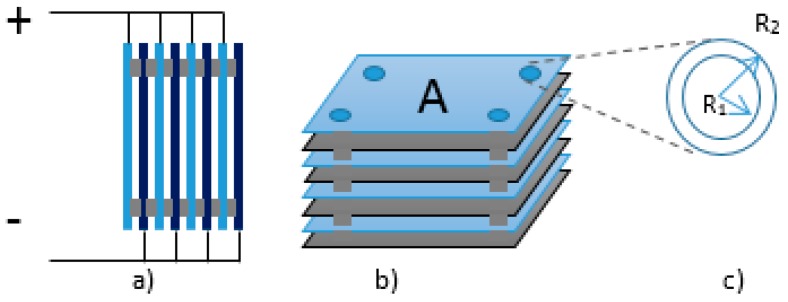
Outline of the capacitive sensor’s prototype. (**a**) Side view; (**b**) Perspective view; and (**c**) Circular dielectric insulators with internal (R_1_) and external (R2) radius.

Figure 2 presents an outline of the sensor’s prototype. The design uses eight plates in parallel, as showed on lateral view of Figure 2a and in the perspective view of Figure 2b. The configuration was adopted with the purpose of increasing the sensor’s capacitance, allowing a better visualization of the capacitance alterations that occur with the variations in temperature, and promote the operation in the stable region of the signal conditioning circuit of the proposed prototype. Zinc plates with 1.0 mm depth form the capacitive sensor. Eight plates of 112.0 cm^2^ (10.0 cm × 11.2 cm) were assembled in parallel and separated by teflon washers (insulators) with a thickness of 2.0 mm. The separation distance between the plates was kept stable by four metallic screws adjusted to room temperature. The diameter of the holes in the plates is 6 mm (R_1_ = 3 mm) and the diameter of the insulators is 1.0 cm (R_2_ = 5 mm), as showed in Figure 2c. The sensor size and the grouping of plates was determined by the capacitance of the order obtained by the sensor that best fit the stability of the signal conditioning circuit. In this case, it is preferred to maintain a large spacing between the plates, allowing better circulation of the fluid during its heating, aiming to favor the uniformity of oil temperature during the measurements.

**Figure 3:**
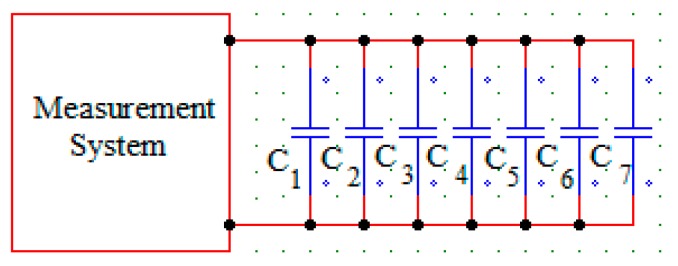
Measuring system of the Capacitive Sensor.

According to Figure 2, the capacitor has eight plates in parallel and insulators between them that provide, together with the screws, the distance between the plates and the coupling. In the area between the plates, we will place the dielectric element and the insulators (four between each pair of plates), decreasing their measuring area. Figure 3 shows a simplified diagram of the arrangement used. As shown in the _illustration_, the set C_1_, C_2_, C_3_, C_4_, C_5_, C_6_ and C_7_ represents a sensor that can be used to characterize the dielectric element kept between its plates or the variations in the dielectric constant as a function of temperature.

The maximum work temperature of the sensor developed is limited to 250 °C. This value was defined because of the melting temperature of the electrical insulation component of the prototype, as indicated in Table 1.

**Table 1:** Materials used in the construction of the sensor and its respective melting temperatures.

Material	Melting Temperature (°C)
Teflon	260 [31]
Zinc	419.5 [32]
Brass	900 [33]

The Equation (1) analysis the behavior of a capacitor at certain temperature. When the sensor is inserted in an environment with significant alterations in the temperature values, the referred equation may be re-written taking into consideration the thermal expansion of the materials employed in the construction of the sensor.

Equation (2) presents the variable capacitance of the sensor, by determining the seven measuring areas between the plates.

(2) $$CV\left(T\right)\;=\;7\cdot \left[\varepsilon_{r1}\left(T\right)\cdot \varepsilon_{0}\cdot \frac{\left(A-4\pi \cdot {R_{1}}^{2}+dA\left(T\right)\right)}{d_{2}+dL\left(T\right)}\right]+Ca$$

Equation (3) presents a fraction of the fixed capacitance (insulators) by determining the measurement of twenty-eight insulators located between the plates.

(3) $$CF(T)\;=7\cdot [4\cdot [\varepsilon_{r2}\left(T\right)\cdot \varepsilon_{0}\cdot \frac{(\pi \cdot ({R_{2}}^{2}-{R_{1}}^{2}))}{d_{2}+dL(T)}]]$$

Thus, the total capacitance C(T) of the sensor is given by the sum of the fractions of the fixed capacitances CF(T) and the variable CV(T) of the sensor

(4) C(T)=CV(T)+CF(T)

In Equations (2) and (3) *dA*(*T*) represents the expansion of the metallic plates area, *dL*(*T*) represents the linear expansion of the insulating spacers between the plates and the screws, $\varepsilon_{r2}\left(T\right)$ is the dielectric constant of the insulating material and, $\varepsilon_{r1}\left(T\right)$ is a non-dimensional function that describes the behavior of the dielectric when submitted to temperature variations. R_1_ is the radius of the plates’ holes, R_2_ is the radium of the insulators and, d is the separation distance between the plates; A is the plates’ area and C(T) is the sensor’s capacitance when submitted to the thermal alterations of the dielectric in question.

By using Equation (4), we calculated the capacitance value with the oil as the dielectric, and using the dielectric permissiveness constant (of the vacuum) ($\varepsilon_{o}$), 8.85 × 10^−12^∙C^2^∙N^−1^∙m^−2^, and at a relative dielectric constant (ɛ_r_), dependent on the dielectric material. For the vegetable oil, we consider $\varepsilon_{r1}$ = 3.5. The capacitance value with these characteristics and space of 2.00 mm between the plates was 1.17 nF. The practical capacitance measurement with the instrument was 1.08 nF. There was, therefore, a difference caused by effect of fringing of the field lines, by the imperfections presented due the materials employed in the construction of the sensor, and by uncertainties due to the used value of the dielectric constant.

Due the ratio between the geometric characteristics to a pair of parallel plate the additional capacitance due effect of fringing of the field lines are smaller than 5%, and not critical to this study due the real goal that are the correlations of parameters. Thus, due to the complex geometry used, we use an adaptive coefficient to estimate this influence and adjust the calculated and the experimentally measured capacitances using an adjustment coefficient or calibration (Ca), found through the Equation (5), determining different coefficients for each dielectric material inserted between the plates.

(5) $$Ca=\frac{C-\left[\left(28\right)\cdot \varepsilon_{r2}\left(T\right)\cdot \varepsilon_{0}\frac{\left(\pi .\left({R_{2}}^{2}-{R_{1}}^{2}\right)\right)}{d_{2}+dL\left(T\right)}\right]}{7\cdot \left[\varepsilon_{0}\frac{\left(A-4\pi \cdot {R_{1}}^{2}+dA\left(T\right)\right)}{d_{2}+dL\left(T\right)}\right].\varepsilon_{r1}\left(T\right)}$$

Thus, we can determine an adjustment coefficient (Ca), to multiply it by the relative dielectric constant. With this procedure, it is possible to decrease the sensor’s reading errors because of the imperfections of the material employed in this construction.

Through Equation (5), the Ca is determined for the vegetable oil, determined as 0.921 with no consideration of the expansion of the material employed.

Therefore, we can re-write Equation (4) by adding the Ca in it and isolating the variable $\varepsilon_{r1}$ (dielectric material between the plates analyzed) which we wish to find, resulting in the following Equation (6).

(6) $$\varepsilon_{r1}\left(T\right)\;=\frac{C-\left[\left(28\right)\cdot \varepsilon_{r2}\left(T\right)\cdot \varepsilon_{0}\cdot \frac{\left(\pi \cdot \left({R_{2}}^{2}-{R_{1}}^{2}\right)\right)}{d_{2}+dL\left(T\right)}\right]}{7\cdot \left[\varepsilon_{0}\cdot \frac{\left(A-4\pi \cdot {R_{1}}^{2}+dA\left(T\right)\right)}{d_{2}+dL\left(T\right)}\right]\cdot Ca}\;$$

The correction Ca used to adjust and correlate experimental values obtained with a theoretical dielectric constant value does not affect the analysis which will be presented, since all experiments are correlated to the experimental values obtained, and evaluation of oil samples is held in functional experimental correlation of physical and chemical tests with the capacitance value, correlated to the relative dielectric constant defined by us.

Between 20 °C and 250 °C was found that, due to dilatation of the sensor and the heating of air, the capacitance increased from 0.41 nF to 0.45 nF. This value is very small compared with the first heating an oil sample, where the capacitance value jumps from 1.09 nF at 20 °C to 10.91 nF at 250 °C, that is, with a growth rate 15 times greater using oil as dielectric rather than air.

### 2.3. Data Acquisition Circuit

**Figure 4:**
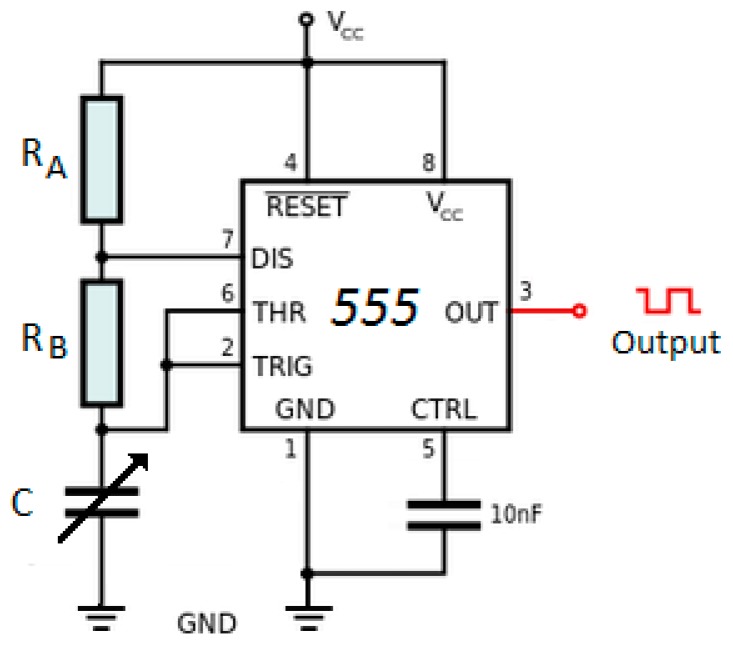
Astable vibrator based on the CI CA555 with frequency depending on a capacitive sensor [16].

An astable vibrator was built to allow the exit frequency to clearly depend on the specific sensor’s capacitance. In order to do that, we used a -CA555 Integrated Circuit [30]. The vibrator’s operation is based on the generation of pulses from the loading and unloading of the capacitor. To determine the duration of these pulses, the high state timing (T_H_), and the low state timing (T_L_), using Equations (7) and (8) [34].

(7) $$T_{L}=0.693\cdot R_{B}\cdot C$$

(8) $$T_{H}=0.693\cdot C\cdot \left(R_{A}+R_{B}\right)$$

where T_H_ is the total time in the high pulse state, [s]; R_B_ is the B resistor illustrated at Figure 4, [Ω]; R_A_ is the resistor A illustrated at Figure 4, [Ω]; T_L_ is the total time in the low pulse state, [s]; and C is the Capacitor, [F].

Thus, when we add Equations (7) and (8), we get to Equation (9), through which it is possible to obtain the sum of the time intervals in high and low states (T), which represents the vibrator’s period (noting that: T = 1/vibrator’s frequency).

(9) $$\text{T}=\text{period}={\text{T}}_{\text{L}}+{\text{T}}_{\text{H}}$$

where T is the total time of the pulse, [s]; T_H_ is the total time in the high pulse state, [s]; e T_L_ is the total time in the low pulse state, [s].

The frequency of the vibrator’s exit pulse, f in Hz units, can be determined by Equation (10), whose magnitudes have been previously presented.

(10) $$\text{f}=\frac{1}{\text{T}}=\frac{1.44}{\left({\text{R}}_{\text{A}}+2{\text{R}}_{\text{B}}\right)\cdot \text{C}}$$

In order to obtain the Reading of a capacitive sensor, we used the adaptation of the capacitive sensor, replacing the standard capacitor in the astable vibrator at Figure 4.

**Figure 5:**
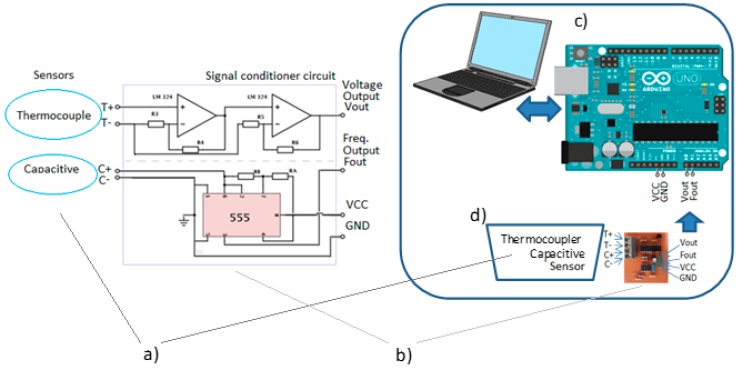
Data acquisition system of the sensors. (**a**) capacitive and temperature sensors; (**b**) Signal conditioner circuit; (**c**) system for storaging data; and (**d**) recipient of oil sample.

To record the data on the capacitive sensor, the circuit shown in Figure 5 was built. The CI CA555 was used to perform the reading of the frequency variation with the Arduino, as the the alteration of the value measured by the sensor occurs.

The resistors used were of 68 Ω for R_A_ and 6.8 kΩ for R_B_. By replacing these values on Equation (10), we can rewrite it as a function of the capacitance. The result is the following Equation (11).

(11) $$\text{C}=\frac{1}{\text{f}\left(0.694{\text{R}}_{\text{A}}+1.388{\text{R}}_{\text{B}}\right)}$$

The timings of the high (T_H_) and low (T_L_) pulses were defined according to the Equations (8) and (9) and calculated to be of 1.94 μs for each one, initially, generating a square frequency wave at a vacuum of 257.7 kHz, obtained for a capacitance value measured experimentally in 0.41 nF.

In order to control the temperature of the soybean oil from 20 °C to 250 °C, we used a K thermocouple, with a measuring window between −200 °C t and 1260 °C [35].

Figure 5 shows the signal conditional circuit used to monitor the signals of capacitance and temperature sensors (Figure 5b), and illustrates the sensors connections (Figure 5a), the system of monitoring of the sensors (Figure 5c) and, the last one, the recipient of the oil sample (Figure 5d).

To validate the results obtained by this circuit, we used variable capacitor of 0.1 nF and 30 nF. This evidence had its measured value by the circuit and also by a Digital Bridge RLC model Gwinstek LCR-819. Subsequently, the measured values were compared with differences of less than 1%. Parallel, a digital thermometer (range of −45 °C to 230 °C) as used to verify the results obtained by the K thermocouple, and the difference between the values obtained by the two sensors is always less than 1%.

## 3. Introduction of Chemical Analysis

The analysis of the quality of the vegetable oil that has been exposed to a heating process involved the determination of a series of chemical indexes [7,9]. In this study, a qualitative analysis of the vegetable oil through the evaluation of the acidity, refraction and iodine indexes were performed in order to find a correlation with the dielectric constant measured by the proposed sensor.

### 3.1. Determination of Acidity in Vegetable Oil

In order to determine the acidity content in the samples of vegetable oil, we performed a triplicate analysis. The samples were weighted and added 25 mL of neutralized ether-alcohol solution (2:1 *v*/*v*) to the sample. The samples were titrated with a standard solution of sodium hydroxide at 0.0100 mol/L. The acidity index was calculated in accordance with Equation (12).

(12) $$INDEX=\frac{28.2\;\cdot v\;\cdot f\cdot \;M}{P}$$

where *v* is the volume (mL) of the sodium hydroxide solution 0.1 mol/L; *f* is the correction factor of the sodium hydroxide solution concentration; and *P* is the weighted sample mass.

#### 3.1.1. Refraction Index

In order to determine the refraction index, we used an Abbe refractometer attached to a water bath at 40 °C. The calibration of the equipment has followed the manufacturer’s instructions, with the use of distilled water.

A small quantity of the sample was added to the lower prism of the equipment with the support of a Pauster pipette. Then, we closed the instrument with the safety lock and waited briefly for the thermal balance between the sample and the instrument. The focus was adjusted for a better reading of the refraction index value. At the end of each sample’s analysis, we cleaned the prisms with soft tissue and ethyl alcohol.

#### 3.1.2. Determination of the Iodine Index

To determine the iodine index, the samples were analyzed in triplicate. The samples were weighted and solubilized with cyclohexane. The Wijs solution was added to the solubilized sample. Next, the solution was left at rest under light at room temperature for 30 min. Then, 10 mL of potassium iodide 15% (*w*/*v*) and 100 mL of deionized water were added. The sample was titrated with sodium thiosulfate solution 0.086 mol/L.

The blank test was performed Equation (13) represents the formula for the calculation of the iodine index of the samples.

(13) $$INDEX=\frac{12.89\cdot \left({\text{V}}_{\text{B}}-{\text{V}}_{\text{A}}\right)\;\cdot \text{M }}{\text{P}}\;$$

where M refers to the molarity of the sodium thiosulfate solution; V_B_ is the volume (mL) spent in the titration of the white; V_A_ is the volume (mL) spent in the titration of the sample; and P is the number of grams of the sample.

#### 3.1.3. Determination of Viscosity

In general, the temperature, composition and type of treatment applied will determine the viscosity of liquid foods [36]. The viscosity of vegetable oils decreases with the increase in temperature due to the breaks of intermolecular bonds via thermal energy. This process facilitates the intermolecular flow, reducing the viscosity of the oil [37]; however, the application temperature cycles may come to change their viscosity characteristics.

The viscosity was measured through the support of a Cup-Ford viscometer. The analysis was based on the oil’s outflow time, recorded in seconds on the first flow interruption. We performed the procedure in duplicate, cleaning all the components between the collections of each sample. Equation (14) represents the equation described in the equipment’s manual for the hole that was used in the experiment [38].

(14) V(t)=3.846·t−17.300

where t represents the time and V(t) the viscosity.

#### 3.1.4. Measurements with Vegetable Oil

In order to analyze the behavior of edible vegetable oils, we proposed a methodology for the analysis of thermal stress in vegetal oils, for example frying, to allow us to analyze possible physical-chemical alterations and correct them with the material’s dielectric constant. To reach such a goal, a capacitive sensor was applied where oil samples were the dielectric element being analyzed, according to the methodology below.

This study presents two types of analysis with soybean oil sample: (i) Heating at constant temperature; and (ii) heating and cooling cycles to the virgin oil.

During the measurements, the sensor was completely submerged in an aluminum recipient containing one liter of soybean oil. The bottom of the recipient containing the sample was heated with a flame, and the temperature went from 20 °C to 250 °C, controlled by thermal sensor. To reduce possible non-homogeneity in heating the oil sample, a stirrer was used during the heating process. The maximum temperature was limited by the melting point of the Teflon insulators used in the construction of the sensor. After the maximum heating temperature, the sample was removed from the flame and the capacitance values were recorded until the moment when the sample temperature returned to room temperature.

At first, one sample of the soybean oil from the same batch was used in a second measurement where an industrial fryer heated the samples. In this measurement, the dielectric variations of the sample were recorded for eight consecutive hours at the constant temperature of 180 °C. After this interval, the sensor readings until the moment when the sample’s temperature returned to room temperature (20 °C).

Next, we performed thermal stress tests, based in cycles. In each cycle, the oil was controlled heating until 250 °C, and after, cooled naturally until the environment temperature. Therefore, for one sample of the oil, we performed eighteen heating measurements with a 24 h interval between each measurement, while trying to maintain the characteristics of the environment. In each case, the capacitance and temperature values were recorded during the complete heating and cooling cycle of the sample. The data were recorded at each 5 °C interval.

**Figure 6:**
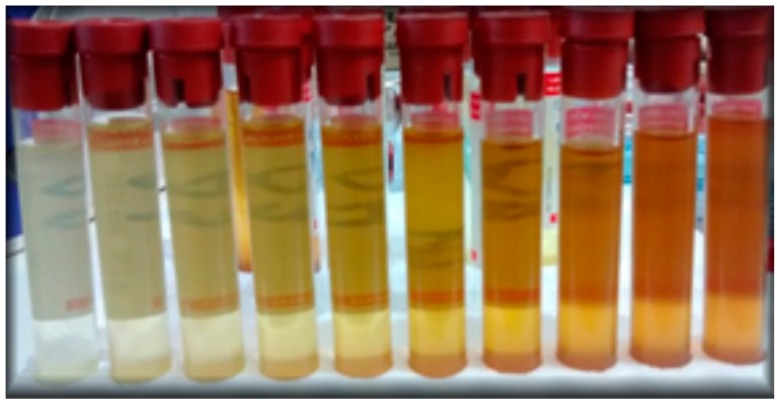
Changes in coloration of oil samples collected after each heating and cooling cycle.

For each heating and cooling cycle, 10 mL of the sample was stored and filed for further measurements, Figure 6, as can be observed the oils color changes after each cycle.

By means of Equation (6), we determined the variations in the dielectric constant resulting from the heating of the samples. To do that, we used the recording of the capacitance value, and took into consideration possible alterations in the sensor during the heating process.

## 4. Results and Discussion

### 4.1. Analysis of the Oil Behavior during Continuous Heating

**Figure 7:**
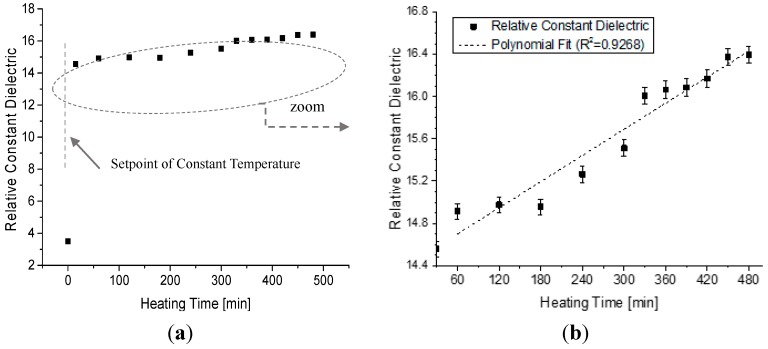
Variation of the Dielectric Constant in relation to time for a soybean oil sample kept at the constant temperature of 180 °C. (**a**) Experimental data of all the heating process since 20 °C; and (**b**) zoom of the data after setpoint of constant temperature.

Figure 7 presents the results of the measurement performed with an electric fryer, where a sample of the virgin oil was exposed to the around constant temperature of 180 °C (±5 °C) for eight consecutive hours. The results shows, after the temperature has stabilized (first minutes), that the dielectric constant suffered changes while all time of the experiment. However, the experimental data showed that the dielectric constant tends to increase proportionally to the time of exposure of the sample kept at a constant temperature. This increase is almost linear as showed by the linear fit, where the calculated coefficient of determination (R^2^) is 0.9268.

### 4.2. Analysis of the Behavior of the Virgin Oil through Thermal Stress in Cycles

**Figure 8:**
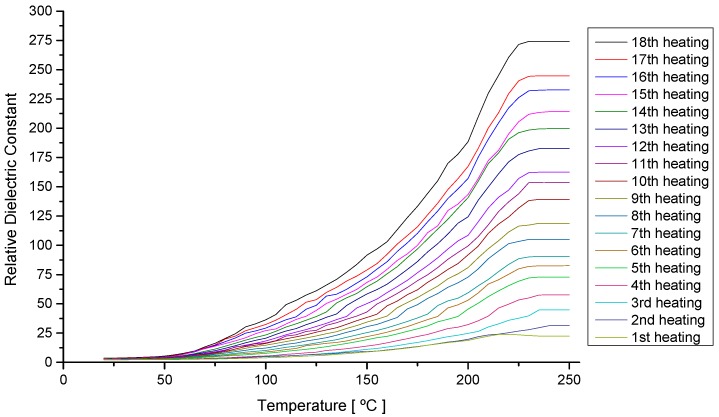
Variation of the Dielectric Constant in relation to the temperature during eighteen heating cycles of a sample of soybean oil (20 to 250 °C).

Figure 8 shows the values of the dielectric constant in relation to the heating temperature of the soybean oil samples (capacitance Reading between 1.08 nF and 135 nF) for each one of the eighteen heating and cooling cycles. In this figure, it is possible to observe that the relative dielectric constant shows increasing values for each heating cycle. This behavior could be attributed to the viscosity decrease, with the increase in temperature [38].

**Figure 9:**
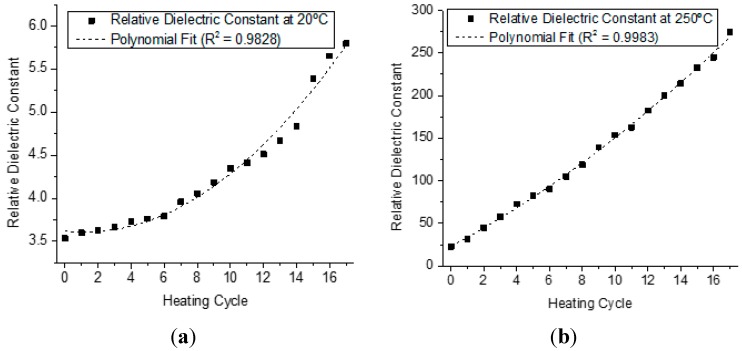
Value of the Dielectric Constant at 20 °C in (**a**) and at 250 °C in (**b**), obtained after each one of the seventeen consecutive heating cycles of the soybean oil samples.

Figure 9 shows the measurements of the dielectric constant at 20 °C in Figure 9a and at 250 °C in Figure 9b, to every time the oil sample was submitted to the heating cycle and returned to room temperature, and the respectives polynomial fits. In these graphs, it is possible to verify that at the end of each cycle, the dielectric constant of the sample suffers a slight increase. These results allow us to conclude that after heating the sample, there is a definite alteration in the capacitance value and, therefore, an irreversible physical-chemical alteration in the samples. It is also possible to conclude that the increase of the temperature works as amplification of the values of the constant dielectric.

Thus, considering that chemical changes can occur during the oil heating, some of the parameters were evaluated in order to compare to the results obteinaed with dieletric constantr values. The acidity, refraction and iodine index were evaluated for each heating and cooling cycle. The acidity index shows the conservation state of oils and fats. The quality parameter indicated for the acceptability of vegetable oils are the lowest possible, as high values indicate significant alterations, which may endanger their use as food or fuel [39].

**Figure 10:**
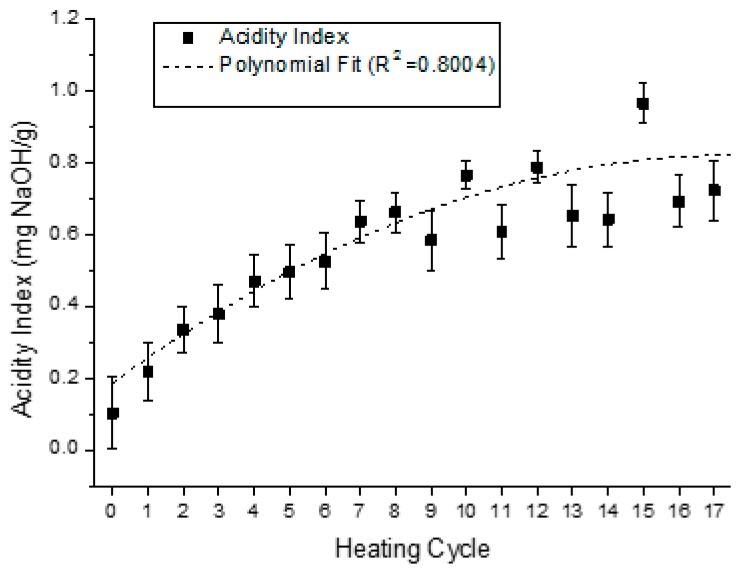
Variation of the acidity index at 20 °C, for each one of the eighteen heating cycles.

For the samples analyzed, the acidity index revealed that at each heating cycle, the acidity index value increases, as showed in the graph at Figure 10. Although a variation on the values of acidity index was observed after the eightieth heating and cooling cycle, there is a clear tendency for the acidity index increasing with heating cycles.

**Figure 11:**
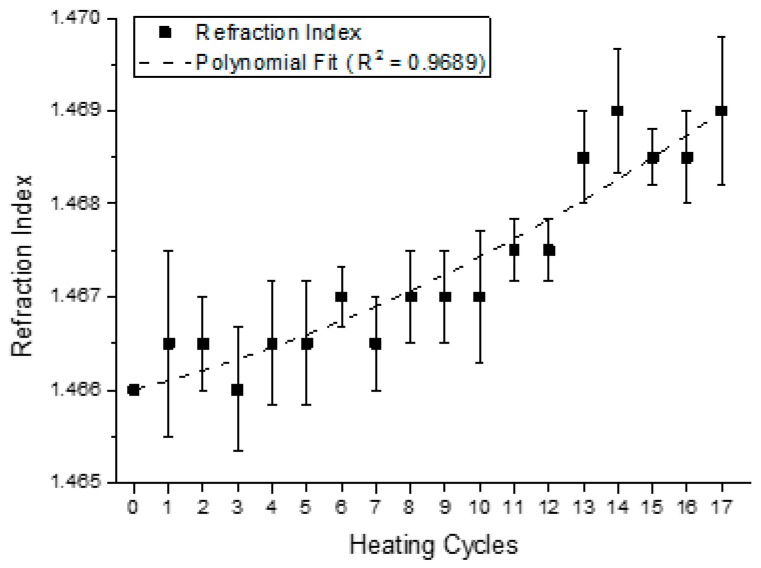
Value of the refraction index at 20 °C, for each one of the consecutive heating cycles.

**Figure 12:**
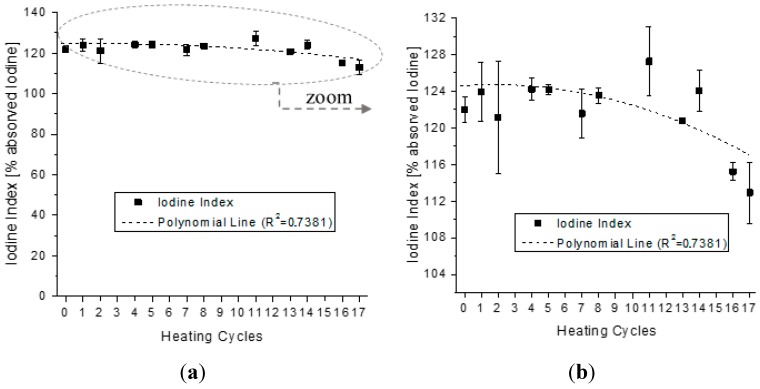
Value of the iodine index at 20 °C, for each one of the seventeen consecutive heating cycles, (**a**) in full scale; and (**b**) zoom on measurement values.

The refraction and iodine indexes, according to the procedures previously described were determined for each sample. The results are presented in Figure 11 and Figure 12, respectively.

The refraction index is defined by the ratio between the speed of light in the air and in the medium (substance under analysis). This value varies according to the alterations in temperature and it tends to increase with the degree of unsaturation of the fatty acids present in the triglycerides [39].

The measures carried out in triplicate values between 1.466 and 1.469 are presented in Figure 11, and the dotted line is the fitting line with coefficient of determination (R^2^) of 0.9689. The obtained results changed slightly with the heating cycles and although a small variation could be observed for the evaluated cycles, the refraction index tends to increase with the number of heating cycles.

The iodine index is a parameter used to foresee the presence of double bonds in a fatty acid ester. The higher the value found for this index, the higher the degree of unsaturation in the sample. The index indicates a tendency of the vegetable oil toward oxidation. The reduction of the iodine index value for the heating cycle is due to the break in the double bond of the unsaturated fatty acids. The measurements of the iodine index occurred at 20 °C [40], and are presented in Figure 12, with the dotted line that is the fitting line with coefficient of determination (R^2^) of 0.7381. The iodine index also showed a slightly tendency to reduction with the heating cycles. The obtained results are in agreements with results of acidity and refraction indexes.

**Figure 13:**
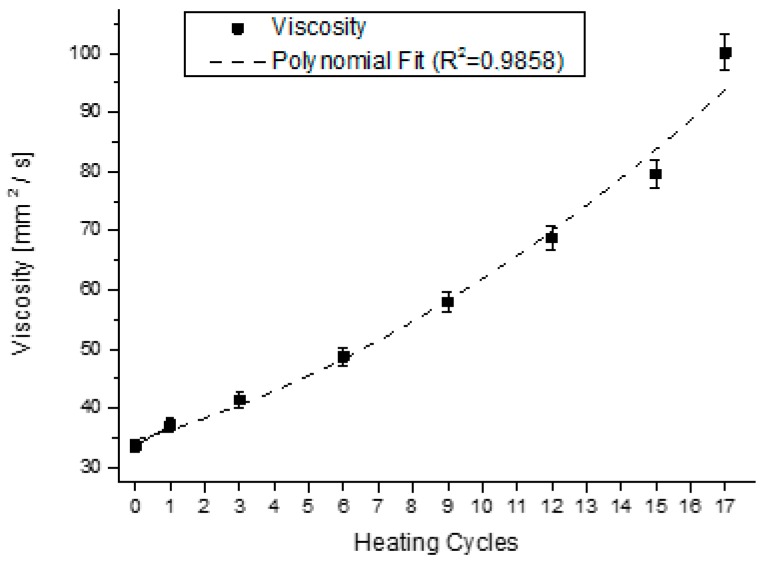
Viscosity of the oil superimposed with the dielectric constant for the samples in Figure 8.

For the samples presented in Figure 8, for the first, third, sixth, nineth, twelfth, fifiteenth and eighteenth heating processes, we performed the viscosity measurements at 20 °C. The results are presented in Figure 13, where the value of viscosity was superimposed with the values of the dielectric constant of the respective samples, and in dotted line, the fitting line with coefficient of determination (R^2^) of 0,9858. It is possible to verify that both indexes present a similar growing behavior.

The equations that describe the behavior of the trend curves of the physical-chemical indexes analyzed at Figure 10 and Figure 13, together with the constant dielectric curve presented in Figure 9, are described in Table 2. The five second-order polynomial fit equations presented refer to the relative dielectric constant, the iodine, refraction, viscosity and acidity indexes of the samples collected.

**Table 2:** Polynomial adjustment referring to the physical-chemical analysis performed.

Parameter	Parameter in Function of T							
Acidity Index	Acidity(T)	=	−	0.0016·T^2^	+	0.0702·T	+	0.1039
Refraction Index	Refr(T)	=		9.0·10^−6^·T^2^	+	1.0·10^−5^·T	+	1.4662
Iodine Index	Iodine(T)	=	−	0.0263·T^2^	−	0.1462·T	+	124.1301
Viscosity	Visco(T)	=		0.1131·T^2^	+	1.4664·T	+	35.067
Relative Dielectric Constant	Er(T)	=		0.0079·T^2^	−	0.0224·T	+	3.5848

The equations reveal that there is a correlation between the behaviors of the different physical-chemical parameters studied. First, all curves were approximate by second-order polynomial equations.

Additionally, to evaluate the fit lines by coefficient (R^2^), we calculate the correlation ratio between the calculated and measured values, using the Pearson Correlation Coefficient (PCC). This ratio indicates how the data are correlated, and has values between −1 and 1, where 1 is an indication of strong positive correlation, −1 is an indication of strong negative correlation, and 0 absence of correlation. Statistically, module values below 0.39 are said weakly correlated, between 0.4 and 0.69 as a correlation moderate, and above 0.7 are said to very strongly correlated [41]. About the order of increasing heating of the samples, we realized that all data collected show strong correlation, as indicated by the values in the Table 3.

**Table 3:** Pearson correlation coefficient to different physico-chemical parameters.

Physico-Chemical Parameter	Coefficient of Determination (R^2^)	Pearson Correlation Coefficient (PCC)
Dielectric Constant	0.9763	0.9466
Acidity Index	0.8004	0.8646
Refraction Index	0.9689	0.9853
Iodine Index	0.7381	0.8616
Viscosity	0.9858	0.9772

**Figure 14:**
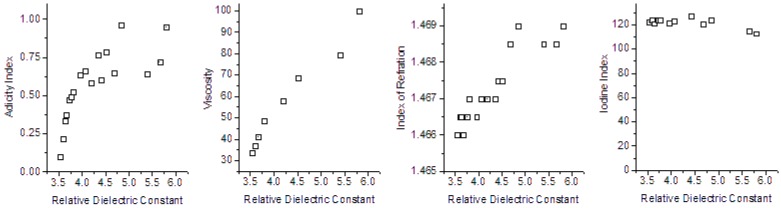
Scatterplots of correlation between Relative Dielectric Constant and the others physico-chemical parameters.

In order to analyze the correlation between the relative dielectric constant and the others four physico-chemical quantities, we present the scatterplots in Figure 14. To each relationship presented, we obtained the Pearson Correlation Coefficient to evaluate the correlation values with each other. The results are presented in Table 4, and shows that there is a hard correlation, because all the PCC are higher than 0.7; and, in special, three of them, higher than 0.9.

**Table 4:** Pearson correlation coefficient to different physico-chemical parameters.

Parameters Correlated			Pearson Correlation Coefficient
Relative Dielectric Constant	*versus*	Acidity Index	0.7255
Relative Dielectric Constant	*versus*	Refraction Index	0.9185
Relative Dielectric Constant	*versus*	Iodine Index	−0.9080
Relative Dielectric Constant	*versus*	Viscosity	0.9880

Is possible to view that all the Pearson Correlation Coefficients are higher than 0.7, representing strong correlation between Relative Dielectric Constant and Acidity Index; and very high correlation between Relative Dielectric Constant and Refraction Index, Iodine Index and Viscosity.

After that, we calculated the standard error of estimative (SEE) for each one parameters, obtain to: SEE_relative dielectric constant_ = 8.3060 × 10^−3^; SEE_acidity index_ = 8.480346 × 10^−3^; SEE_iodine index_ = 12.2598; SEE_viscosity_ = 9.7597; and SEE_index of refraction_ = 1.1032 × 10^−7^. To analyze and validate these values, the hypothesis validation test with the distribution F, based on the F-observed and F-critical values, was calculated.

This F-critical value is based on level of significance. If the obtained F-observed value is equal to or larger than this critical F-value, then the result is significant at that level of probability [41]. Based on this, viscosity, index of refraction, acidity index and relative dielectric constant had an interval of confidence higher than 99%, while the iodine index, higher than 92%, which validates the approaches statistically.

Considering those correlations, we can conclude that the possibility exists that oil may have its physico-chemical characteristics determined through the correlation with the dielectric constant, which we can measure for a much lower cost than the physical-chemical analyses.

In particular, since the acidity index value is one of the main parameters used to evaluate the state of degradation of an oil due to thermal stress caused during its use in homes and restaurants; we propose that, since this index correlated to the dielectric constant, that this can be an efficient parameter and inexpensive method to classify samples of used oil, since the traditional physico-chemical analysis only can be performed in specialized laboratories.

## 5. Conclusions

Several analyses of the dielectric constant variation have been performed for soybean oil samples during eighteen cycles of heating and cooling of the room temperature up to the temperature of 250 °C. Other oils were analyzed during a prolonged cycle (eight continuous hours) of heating at a constant temperature (180 °C). The results show that the dielectric constant increases with the temperature increase. At the maximum heating temperature, which has been proposed for the eighteen heating processes of the virgin soybean oil, for example, at 250 °C, the dielectric constant increased one hundred times its initial value. After each complete heating and cooling cycle, we verified that the dielectric constant value suffers a slight addition, indicating a definite alteration in the physical-chemical properties of the samples. The results obtained suggest a simple method to qualify the state of vegetable oils consinuously submitted to temperature variations.

In the food industry, this test can be sufficient to analyze the quality of the soybean oil used to fry foods in a simple, fast and accessible manner. However, in order to do that, the experiment needs to correlate the behavior observed with a combination of the analysis presented and the physical-chemical analysis which allows the determination of a limit value for the dielectric constant, based on which the sample is degraded for feeding purposes. It also allows us to define where this material may be best reapplied as a biofuel, painting or other applications.

In order to correlate such results for the dielectric constant, we performed the analysis of acidity, refraction, viscosity and iodine levels for each sample collected in consecutive heating cycle. We report a strong correlation in the behaviour of oil parameters in this process between Pearson Correlation Coeficient.

This study concluded that the higher the value of the dielectric constant found for the oil analyzed, the higher the deterioration of the oil sample (in relation to the physico-chemical parameters that have been analyzed). Additionally, we can conclude that it is more easy quantify this deterioration in high temperatures, due the amplification of the values of the dielectric constant.
